# Brassinosteroid Synthesis and Perception Differently Regulate Phytohormone Networks in *Arabidopsis thaliana*

**DOI:** 10.3390/ijms26199644

**Published:** 2025-10-02

**Authors:** Yaroslava Bukhonska, Michael Derevyanchuk, Roberta Filepova, Jan Martinec, Petre Dobrev, Eric Ruelland, Volodymyr Kravets

**Affiliations:** 1 V.P. Kukhar Institute of Bioorganic Chemistry and Petrochemistry, National Academy of Sciences of Ukraine, 02094 Kyiv, Ukraine; yaroslavabukhonska@gmail.com (Y.B.); derevmyk@ukr.net (M.D.); 2 Institute of Experimental Botany of the Czech Academy of Sciences, 16502 Prague, Czech Republic; filepova@ueb.cas.cz (R.F.); martinec@ueb.cas.cz (J.M.); dobrev@ueb.cas.cz (P.D.); 3 Université de Technologie de Compiègne, UPJV, CNRS, Génie Enzymatique et Cellulaire, 60203 Compiègne, France; eric.ruelland@utc.fr

**Keywords:** brassinosteroid, 24-epibrassinolide, brassinazole, phytohormones, metabolic regulation, *Arabidopsis thaliana*

## Abstract

Brassinosteroids (BRs) are essential regulators of plant development and stress responses, but the distinct contributions of BR biosynthesis and signaling to hormonal crosstalk remain poorly defined. Here, we investigated the effects of the BR biosynthesis inhibitor brassinazole (BRZ) and the BR-insensitive mutant *bri1-6* on endogenous phytohormone profiles in *Arabidopsis thaliana*. Using multivariate analysis and targeted hormone quantification, we show that BRZ treatment and BRI1 disruption alter hormone balance through partially overlapping but mechanistically distinct pathways. Principal component analysis (PCA) and hierarchical clustering revealed that BRZ and the *bri1-6* mutation do not phenocopy each other and that BRZ still alters hormone profiles even in the *bri1-6* mutant, suggesting potential BRI1-independent effects. Both BRZ treatment and the *bri1-6* mutation tend to influence cytokinins and auxin conjugates divergently. On the contrary, their effects on stress-related hormones converge: BRZ decreases salicylic acid (SA), jasmonic acid (JA), and abscisic acid (ABA) in the WT leaves; similarly, *bri1-6* mutants show reduced SA, JA, and ABA. These results indicate that BR biosynthesis and BRI1-mediated perception may contribute independently to hormonal reprogramming, with BRZ eliciting additional effects, possibly via metabolic feedback, compensatory signaling, or off-target action. Hormone correlation analyses revealed conserved co-regulation clusters that reflect underlying regulatory modules. Altogether, our findings provide evidence for a partial uncoupling of BR levels and BR signaling and illustrate how BR pathways intersect with broader hormone networks to coordinate growth and stress responses.

## 1. Introduction

Brassinosteroids (BRs) are essential plant steroid hormones that regulate a wide range of physiological processes, including cell elongation, vascular differentiation, reproductive development, and stress responses [1,2,3]. BRs play a key role in helping plants cope with both biotic and abiotic stresses, such as drought [4,5,6,7], salinity [8,9,10], and pathogen attack [11,12,13]. Their involvement in stress responses is particularly significant as they can modulate stomatal closure during water stress, activate defense mechanisms against pathogens, and promote recovery from oxidative stress [14]. Importantly, BRs regulate stress-induced gene expression and protein synthesis that support plant survival under unfavorable conditions [15]. Contrary to previous assumptions, BRs are present not only in the Brassicaceae family but are widely distributed across the plant kingdom, from dicots to monocots, including crop species like rice and maize.

BRs are derived from campesterol through a complex biosynthetic pathway involving multiple enzymes, including DWARF4 (DWF4), CPD (CONSTITUTIVE PHOTOMORPHOGENIC DWARF), and BR6ox (BRASSINOSTEROID-6-OXIDASE) [16,17]. The final bioactive BRs, such as brassinolide (BL) and 24-epibrassinolide (EBL), are produced in various plant tissues. However, their distribution and transport within the plant cells remain an area of active investigation. Contradictory findings have been reported regarding the specific transport mechanisms, with some studies suggesting rapid movement through the phloem, while others propose limited long-distance transport [18,19]. Inhibition of BR biosynthesis, for example, using brassinazole (BRZ), disrupts plant growth and development, leading to phenotypes such as dwarfism and reduced stress tolerance [20,21,22]. BRZ specifically targets DWF4 and other key enzymes, including CPD and BR6ox, blocking the production of endogenous BRs and providing a powerful tool to study their biological roles [22,23].

BR perception and signal transduction are mediated by a well-defined receptor complex at the plasma membrane. The BRASSINOSTEROID INSENSITIVE 1 (BRI1) receptor kinase, along with its co-receptor BAK1 (BRI1-ASSOCIATED KINASE 1)**,** initiates the signaling cascade upon BR binding [24,25]. This activates a downstream phosphorylation cascade that ultimately regulates the BZR (BRASSINAZOLE-RESISTANT) family of transcription factors, including BZR1 and BES1 (BRI1-EMS-SUPPRESSOR 1) [26,27]. Phosphorylation of BZR1 and BES1 leads to their activation and nuclear translocation, where they modulate the expression of BR-responsive genes that influence processes such as cell expansion, stress adaptation, and reproductive development [28]. Mutations in BRI1, such as the *bri1-6* allele, impair BR perception and result in severe developmental defects, including dwarfism and altered hormone homeostasis [29,30]. The *bri1-6* allele is a T-DNA insertion mutation that leads to loss of function of the BRI1 receptor, which impairs BR signaling and leads to altered plant morphology.

While the core components of BR synthesis and signaling are well understood, their interactions with other phytohormones—such as abscisic acid (ABA), auxins, cytokinins, jasmonic acid (JA), and salicylic acid (SA)—remain an area of active research. BRs are known to antagonize ABA signaling [31,32] and modulate JA and SA pathways [33,34], but the precise mechanisms of these interactions, particularly under non-stress conditions, are not fully elucidated.

In this study, we investigated how exogenous application of epibrassinolide (EBL) and inhibition of BR biosynthesis with brassinazole (BRZ) influence the endogenous content of key phytohormones in *Arabidopsis thaliana* wild-type (WT) plants and the *bri1-6* mutant. Our goal was to clarify how BR availability and BR signaling via BRI1 integrate with broader hormonal networks. Taken together, our work reveals that BRI1 is a key component in coordinating hormonal cross-regulation but also highlights that BR availability alone, induced by BRZ, can alter hormone levels in a BRI1-independent manner. This distinction provides new insights into how BRs modulate hormonal crosstalk to maintain the balance between growth and stress adaptation.

## 2. Results

### 2.1. Overall Survey of the Effect of EBL and BRZ on the Two Genotypes

**Figure 1:**
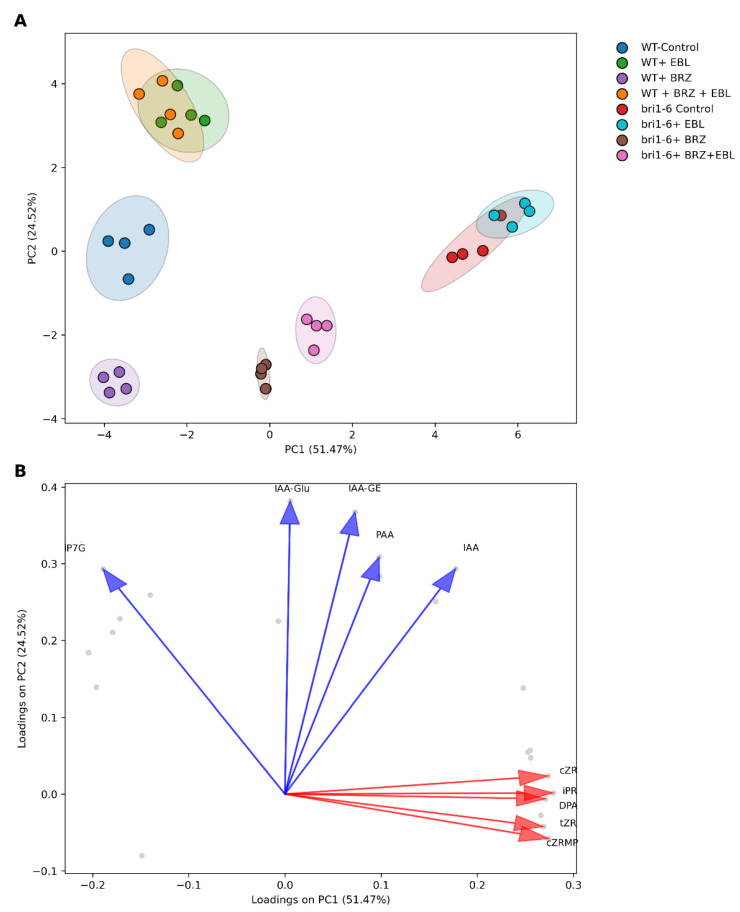
Principal Component Analysis (PCA) and Variable Contributions. (**A**) PCA plot showing the distribution of samples based on the first two principal components (PC1 and PC2). Each point represents a sample, colored by group. Ellipses indicate the 95% confidence interval for each group. (**B**) Contribution plot of the top 5 variables to PC1 and PC2. Red arrows and labels represent variables contributing most to PC1, while blue arrows and labels represent variables contributing most to PC2. The arrows indicate the direction and magnitude of each variable’s contribution to the principal components. Grey dots represent all variables projected into the PC1–PC2 space, while colored arrows highlight the top 10 contributors to variance along PC1 and PC2 axes.

We provide a comprehensive overview of the hormone profile responses to exogenous brassinolide (EBL, 10 nM) and the BR biosynthesis inhibitor brassinazole (BRZ, 1 µM) in two *Arabidopsis thaliana* genotypes: wild-type (WT) and the *bri1-6* mutant. The PCA scores plot (Figure 1A) shows sample distributions along the first two principal components (PC1: 51.47%, PC2: 24.52%), together explaining 75.99% of the total variance. The biosynthetic and/or catalytic pathways depicting the hormones and the metabolites we detected are displayed as Supplementary File S1 [35,36,37,38,39,40]. In WT plants, WT-control (blue circles) and WT + EBL (green circles) are clearly separated along PC2, with WT + EBL shifted positively. In contrast, WT + BRZ (purple circles) is shifted toward the negative side of PC2, indicating a metabolic profile that opposes that of WT + EBL, consistent with BRZ antagonizing BR-dependent metabolic responses. The WT + EBL + BRZ (orange circles) group overlaps with WT + EBL. In the *bri1-6* mutant, all groups are clearly separated from WT groups along PC1. The *bri1-6* control (red circles) and *bri1-6* + EBL (cyan) groups overlap closely, confirming a lack of EBL response in the absence of BRI1. However, the *bri1-6* + BRZ (brown circles) group is shifted negatively along PC2 compared to the *bri1-6* control, suggesting that BRZ still modulates hormone levels independently of BRI1. The *bri1-6* + EBL + BRZ (pink circles) group is located near *bri1-6* + BRZ but remains distinguishable.

The contribution plot (Figure 1B) identifies key hormones driving the separation along PC1 and PC2. Cytokinins (e.g., cZR, tZR, iPR, cZRMP) contribute primarily to PC1, while auxin-related metabolites, such as IAA and IAA-Glu, are positively associated with PC2.

**Figure 2:**
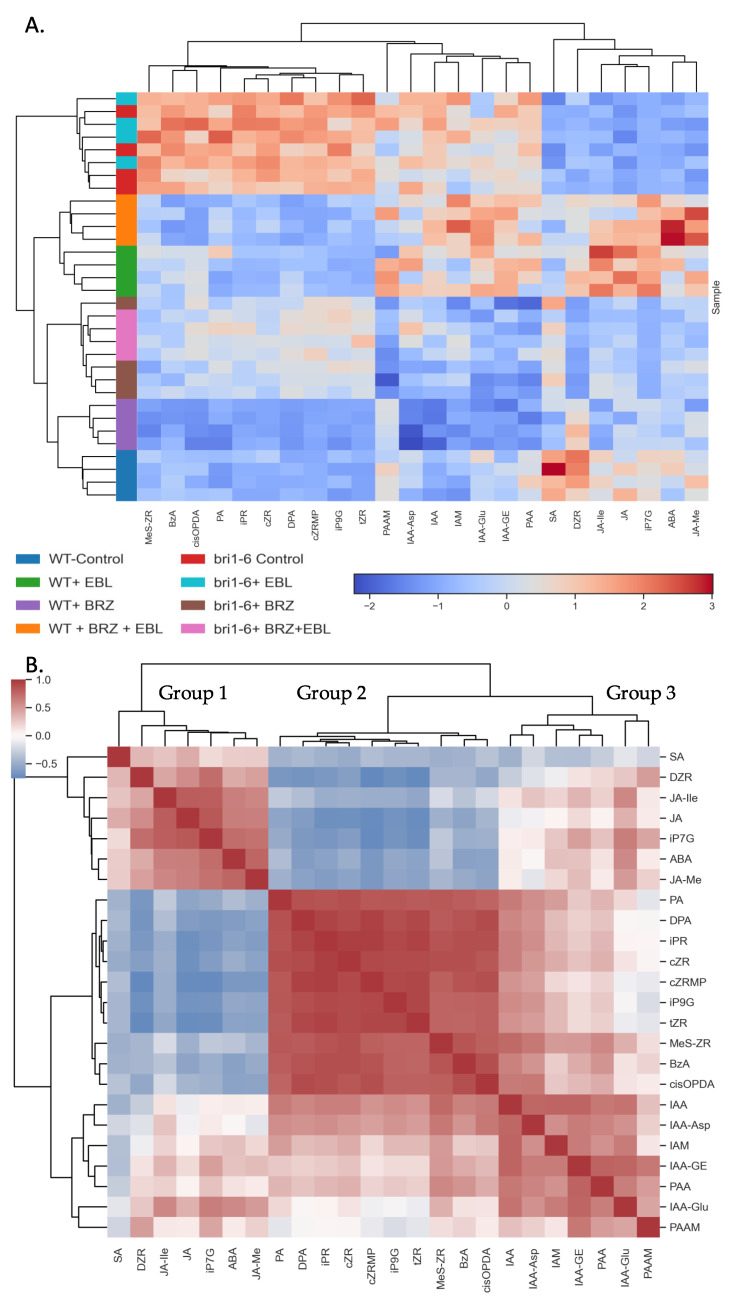
(**A**). Hierarchical biclustering of samples (rows) and variables (columns), based on Z-score–normalized values of the measured metabolites across all conditions. Row colors indicate sample group identity. The color scale reflects Z-scores (mean-centered, unit variance), with red and blue indicating high and low relative abundance, respectively. (**B**). Hierarchical clustering of the Pearson correlation matrix among variables. Variables with similar abundance profiles across samples cluster together, revealing co-regulated or functionally related metabolite groups. Clustering was performed using average linkage and Euclidean distance on the correlation matrix.

The heatmap (Figure 2A) displays distinct hormone profiles across treatments. WT groups show clear differences between control, BRZ, and EBL treatments. In *bri1-6*, hormone patterns differ markedly from WT, and BRZ still induces shifts even in the mutant background, supporting a partial uncoupling of BR levels and BR signaling.

The correlation heatmap (Figure 2B) reveals three hormone clusters. Group 1 includes SA, DZR, JA, JA-Ile, JA-Me, iP7G, and ABA. Group 2 contains PA, DPA, iPR, CZR, CZRMP, IP9G, MES-ZR, BzA, and cis-OPDA. Group 3 comprises IAA, IAA-Asp, IAM, IAA-GE, IAA-Glu, PAA, and PAAM. Group 1 hormones show a negative correlation with Group 2 and a positive correlation with Group 3 (except for SA, which is negatively correlated with Group 3). Group 2 and Group 3 hormones are positively correlated with each other.

### 2.2. The Impact of EBL and Brassinazole (BRZ) on ABA and Its Metabolites

**Figure 3:**
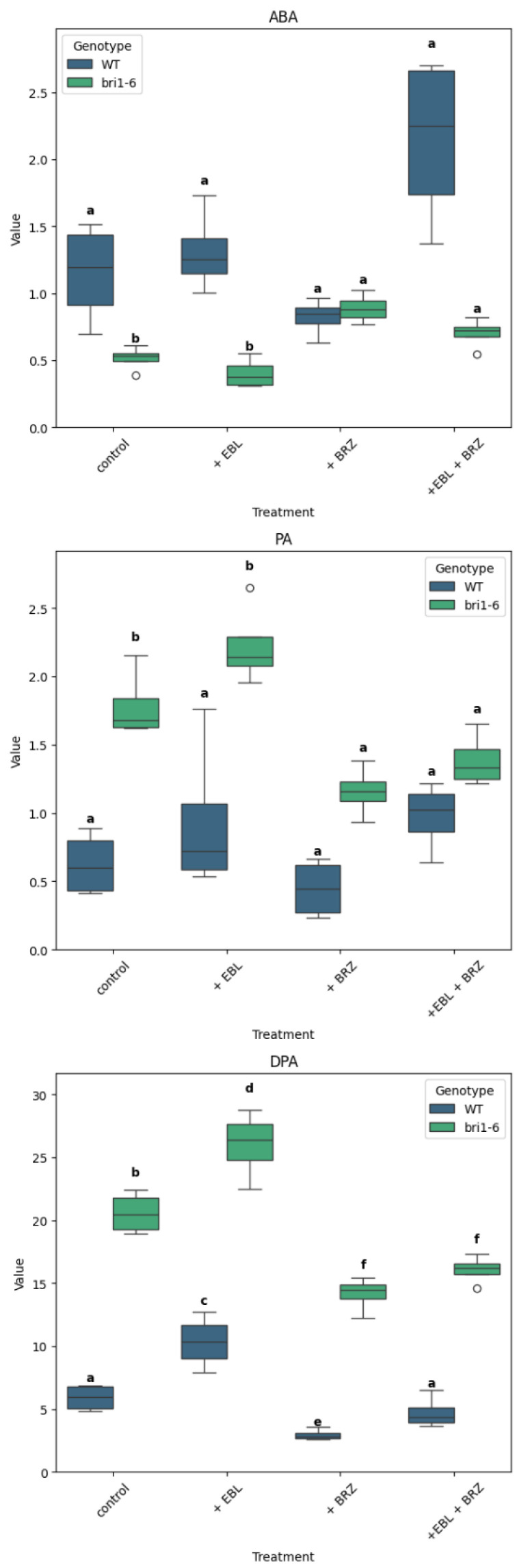
Distribution of ABA, PA, and DPA levels across treatments and genotypes. Boxplots show the levels of ABA, PA, and DPA for wild-type (WT) and *bri1-6* plants under four treatments: control, +EBL, +BRZ, and +EBL + BRZ. Each boxplot represents the distribution of four biological replicates per condition. Letters above the boxplots indicate statistical grouping based on Fisher’s LSD post hoc test (α = 0.05): groups sharing the same letter are not significantly different, while different letters indicate significant differences; the circle on the graph represents an outlier. The “viridis” color palette distinguishes genotypes (WT in blue, *bri1-6* in green). The y-axis is scaled to the maximum value of each compound for clarity.

We now consider each hormone type in more detail, starting with ABA and its metabolites (Figure 3). In the control plants, the ABA content was relatively low at 1.15 ± 0.19 pmol/g fresh weight; its metabolite PA was 0.63 ± 0.12 pmol/g fresh weight; the DPA content was higher at 5.91 ± 0.53 pmol/g fresh weight.

In WT plants, ABA levels were not significantly altered in response to EBL or to BRZ. In contrast, the *bri1-6* mutant maintained consistently lower ABA levels across all treatments than WT. While EBL had no effect on ABA level, BRZ did increase ABA in *bri1-6*, which is confirmed by +EBL + BRZ treatment.

PA data exhibits a reverse pattern. WT plants showed lower PA levels across treatments compared to *bri1-6*. In WT, the treatments with EBL or/and BRZ did not lead to significant level changes. In *bri1-6*, the treatment with BRZ led to a decrease in PA, which is confirmed in the +EBL + BRZ treatment.

As for DPA, the pattern is the same for WT and *bri1-6* plants: a higher accumulation with EBL compared to control, a decreased accumulation in response to BRZ. The DPA level was consistently lower in WT than in *bri1-6* plants.

### 2.3. The Impact of EBL and Brassinazole (BRZ) on SA and Its Metabolites

**Figure 4:**
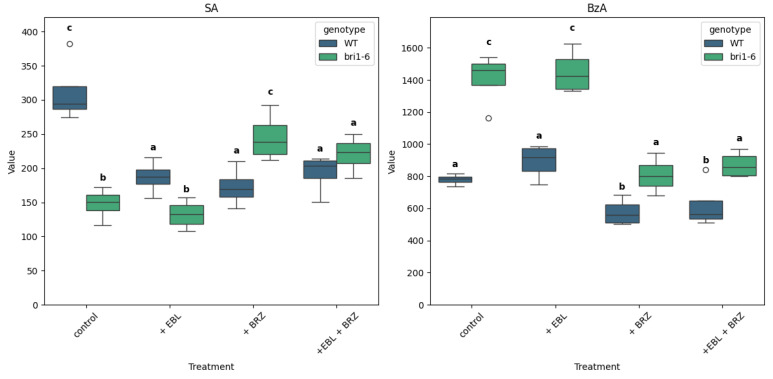
Levels of SA and BzA across treatments and genotypes. Boxplots show the distribution of SA and BzA for wild-type (WT) and *bri1-6* plants under four treatments: control, +EBL, +BRZ, and +EBL + BRZ. Each boxplot represents the distribution of four biological replicates per condition. Values are expressed in pmol/g FW. Letters above the boxplots indicate statistical grouping based on pairwise *t*-tests (Fisher’s LSD, α = 0.05): groups sharing the same letter are not significantly different, while different letters indicate significant differences; circles on the graph represent outliers. The “viridis” color palette distinguishes genotypes (WT in blue, *bri1-6* in green). The y-axis is scaled to the maximum value of each compound for clarity.

In WT plants, the application of EBL led to a decrease in SA content from 311.5 pmol/g FW to 186.65 pmol/g FW (Figure 4). Surprisingly, BRZ treatment also reduced SA levels compared to the control. In *bri1-6* plants, treatment by EBL does not modify SA level, while treatments with BRZ alone or with EBL lead to higher SA levels. Note that the control SA level in *bri1-6* plants is significantly lower than in the WT.

Benzoic acid (BzA) is a precursor of SA. Its level was higher in *bri1-6* leaves compared to WT. EBL treatment had no effect on BzA level, neither in WT nor in *bri1-6* leaves. BRZ treatment reduced BzA levels in WT and *bri1-6* leaves; combined treatment with BRZ and EBL did not change BzA content compared to BRZ alone.

### 2.4. The Impact of EBL and Brassinazole (BRZ) on JA and Related Metabolites

**Figure 5:**
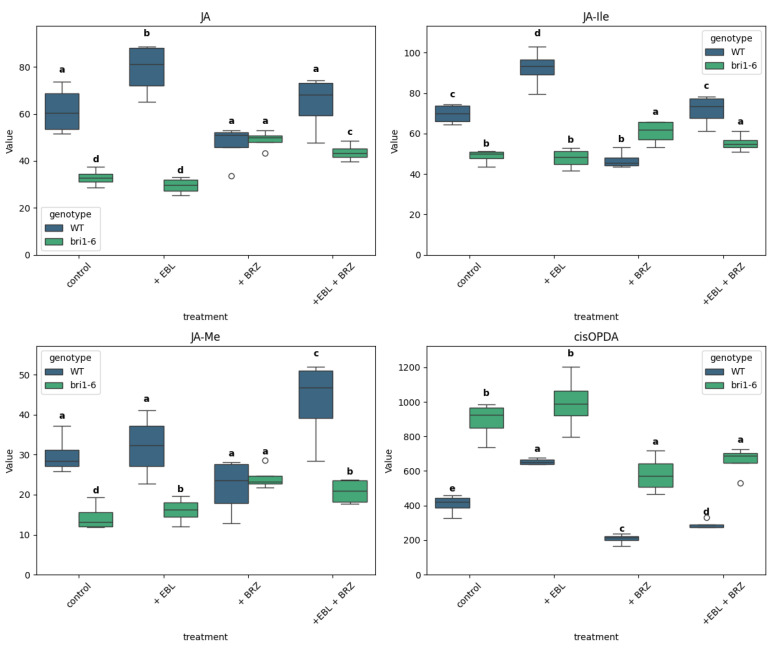
Levels of jasmonate metabolites across treatments and genotypes. Boxplots show the distribution of JA, JA-Ile, methyl jasmonic acid (JA-Me), and cis-12-oxo-phytodienoic acid (cis-OPDA) for wild-type (WT) and *bri1-6* plants under four treatments: control, +EBL, +BRZ, and +EBL + BRZ. Each boxplot represents the distribution of four biological replicates per condition. Values are expressed in pmol/g FW. Letters above the boxplots indicate statistical grouping based on pairwise *t*-tests (Fisher’s LSD, α = 0.05): groups sharing the same letter are not significantly different, while different letters indicate significant differences; circles on the graph represent outliers. The “viridis” color palette distinguishes genotypes (WT in blue, *bri1-6* in green). The y-axis is scaled to the maximum value of each compound for clarity.

EBL treatment increased the levels of jasmonic acid (JA) in WT plants; the treatment with BRZ did not alter the JA level (Figure 5). In the *bri1-6* leaves, EBL has no effect; BRZ leads to an increase in JA that we observe also in the combined BRZ + EBL treatment.

A similar pattern is observed for JA-Ile. In WT plants, EBL treatment leads to an increased accumulation, but for JA-Ile, BRZ leads to a decreased accumulation compared to the control. With the combined BRZ + EBL treatment, the JA-Ile level is similar to that in the control. In *bri1-6* mutant leaves, EBL treatment has no effect; BRZ leads to a small increase, also observed in the combined BRZ + EBL treatment.

For JA-Me, the treatments did not lead to altered accumulation in the WT leaves, but for the combined BRZ + EBL one, that leads to an increased level compared to the control. In *bri1-6* mutant leaves, EBL treatments lead to a small increase, BRZ to a higher increase, but the combined BRZ + EBL treatment leads to a similar accumulation as in EBL treatment alone.

As for the content of cis-OPDA, it is increased by EBL treatment but decreased by BRZ treatment in WT leaves. In *bri1-6* plants, the effect of EBL is abolished while the effect of BRZ is still observed; it is also visible in the combined BRZ + EBL treatment.

Note that the JA level, but also the JA-Ile and JA-Me levels, are higher in WT versus *bri1-6* leaves in control conditions. On the contrary, the cis-OPDA level in WT is lower than in *bri1-6* leaves.

### 2.5. The Impact of EBL and Brassinazole (BRZ) on Auxins

**Figure 6:**
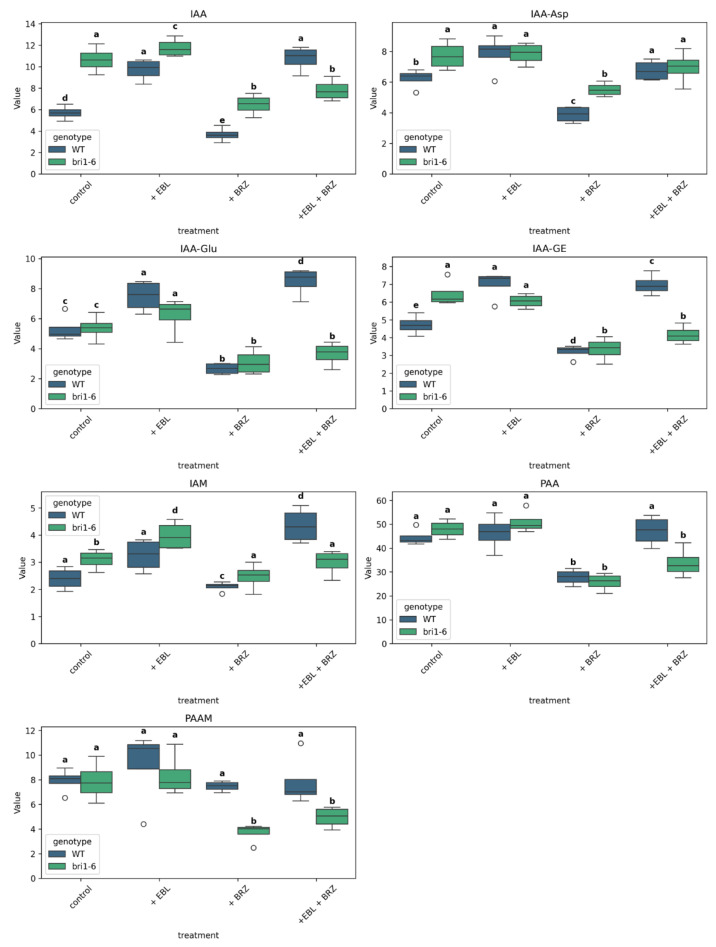
Boxplots show the distribution of IAA and its metabolites IAA-aspartate (IAA-Asp), IAA-glutamate (IAA-Glu), IAA-glucose ester (IAA-GE), indole-3-acetamide (IAM), phenylacetic acid (PAA), and phenylacetamide (PAAM) in *Arabidopsis thaliana* WT and *bri1-6* plants under four treatments: control, +EBL, +BRZ, and +EBL + BRZ. Each boxplot represents the distribution of four biological replicates per condition. Values are expressed in pmol/g FW. Letters above the boxplots indicate statistical grouping based on pairwise *t*-tests (Fisher’s LSD, α = 0.05): groups sharing the same letter are not significantly different, while different letters indicate significant differences; circles on the graph represent outliers. The “viridis” color palette distinguishes genotypes (WT in blue, *bri1-6* in green). The y-axis is scaled to the maximum value of each metabolite for clarity.

In our investigation, we measured the content of indole-3-acetic acid (IAA) and its metabolites in the leaves of *Arabidopsis thaliana* WT and *bri1-6* mutant plants (Figure 6).

For IAA, the treatment with EBL resulted in an increase in IAA in WT leaves, while the treatment with BRZ led to a decrease in IAA when compared to the control. In the combined BRZ + EBL treatment, a level comparable to the one attained with EBL treatment is observed. In *bri1-6* mutant plants, the effect of EBL alone is abolished; the decrease by BRZ is still observed and is also observed in the combined BRZ + EBL treatment.

The same pattern can be seen with IAA-Asp, but for the combined BRZ + EBL treatment in the *bri1-6* mutant leaves, for which the accumulation obtained is not different from that in the control.

The pattern for IAA-Glu is similar to that of IAA, but for the EBL treatment in the *bri1-6* mutant leaves, that could still be considered as increased compared to the level in the *bri1-6* control. The pattern for IAA-GE is similar to that of IAA. For IAM, PAA, and PAAM, EBL has no effect in WT leaves. BRZ leads to a decreased level for IAM and PAA in WT leaves, a decrease abolished in the combined BRZ + EBL treatment. A decrease in IAM, PAA, and PAAM levels is observed in response to BRZ in *bri1-6* leaves, confirmed in the combined BRZ + EBL treatment.

In control conditions, the levels of IAA, IAA-Asp, IAA-GE, and IAM are higher in *bri1-6* mutant leaves than in WT leaves.

### 2.6. The Impact of EBL and Brassinazole (BRZ) on Cytokinins

**Figure 7:**
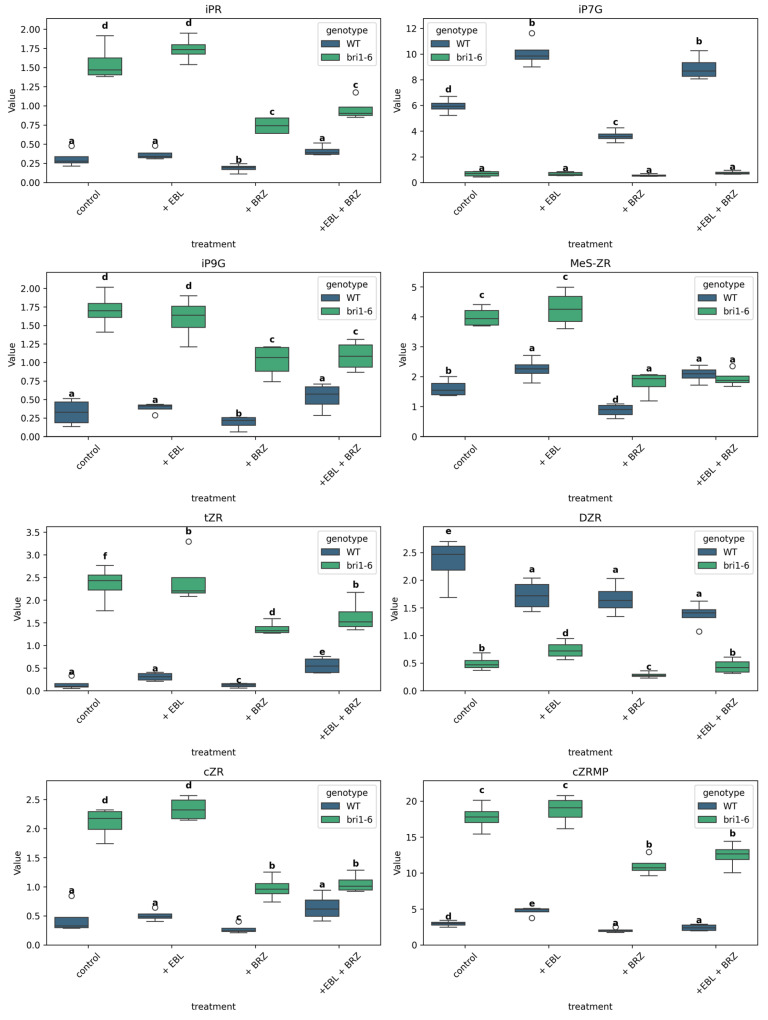
Boxplots show the distribution of isopentenyl adenine, isopentenyl adenine-7-glucoside (iP7G), isopentenyl adenine-9-glucoside (iP9G), 2-methylthio zeatin ribosides of trans-zeatin, cis-zeatin (MeS-ZR), trans-zeatin riboside (tZR), dihydrozeatin riboside (DZR), cis-zeatin riboside (cZR), and cis-zeatin riboside monophosphate (cZRMP) in *Arabidopsis thaliana* wild-type (WT) and *bri1-6* plants under four treatments: control, +EBL, +BRZ, and +EBL + BRZ. Each boxplot represents the distribution of four biological replicates per condition. Values are expressed in pmol/g FW. Letters above the boxplots indicate statistical grouping based on pairwise *t*-tests (Fisher’s LSD, α = 0.05): groups sharing the same letter are not significantly different, while different letters indicate significant differences; circles on the graph represent outliers. The “viridis” color palette distinguishes genotypes (WT in blue, *bri1-6* in green). The y-axis is scaled to the maximum value of each metabolite for clarity.

We analyzed the effects of EBL and BRZ treatment on key CK fractions, focusing on isopentenyl adenine (iP), isopentenyl adenine-7-glucoside (iP7G), isopentenyl adenine-9-glucoside (iP9G), a mixture of 2-methylthio-zeatin ribosides of trans-zeatin and cis-zeatin (MeS-ZR), as well as trans-zeatin riboside (tZR), dihydrozeatin riboside (DZR), cis-zeatin riboside (cZR), and cis-zeatin riboside monophosphate (cZRMP) (Figure 7).

In WT plants, the level of iPR, iP9G, tZR, and cZR did not change significantly when treated with EBL. On the contrary, EBL increased the accumulation of iP7G, MeS-ZR, and CZRMP in WT plants. Those increased accumulations are not observed in *bri1-6* mutant leaves. BRZ treatment led to a decreased accumulation of all the assessed molecules in WT plants. The decreased accumulation is also observed for all molecules but iP7G in the *bri1-6* leaves. The accumulation observed in the combined BRZ + EBL treatment is comparable to that with BRZ alone in *bri1-6* leaves for all molecules but tZR and DZR. In WT plants, the accumulation observed in the combined BRZ + EBL treatment is most often higher than that with BRZ alone, but for iP7G, DZR, and CZRMP.

When compared to WT leaves, *bri1-6* leaves had a higher basal level of iPR, iP9G, MeS-ZR, tZR, cZR, and cZRMP, but a lower level of DZR and iP7G.

## 3. Discussion

Our findings provide significant insights into how brassinosteroid (BR) synthesis and perception regulate hormonal crosstalk in *Arabidopsis thaliana*. By analyzing the hormone profiles of wild-type (WT) and *bri1-6* mutant leaves treated with epibrassinolide (EBL), brassinazole (BRZ), or both, we dissected the respective contributions of BR biosynthesis and BR signaling via BRI1. These results extend previous work on BR-mediated hormonal regulation [15,33], revealing an uncoupling between BR levels and receptor-dependent signaling.

Although several BR biosynthesis mutants such as *dwf4*, *cpd*, and *br6ox1/2* have been described [16], their severe dwarfism, sterility, and pleiotropic developmental defects make them less suitable for comparative hormone profiling. In contrast, BRZ treatment allows reversible, tunable inhibition of BR biosynthesis without the confounding effects of long-term developmental reprogramming and was thus used in this study.

PCA and hierarchical clustering analyses clearly showed that BRZ and the *bri1-6* mutation do not phenocopy each other, despite both conditions being supposedly associated with reduced BR signaling output. While EBL shifted WT profiles positively along PC2, the *bri1-6* mutation shifted them positively along PC1. BRZ moved the WT data negatively in PC2, consistent with the inhibiting role of BRZ on endogenous BR and therefore an opposite role to EBL [20,22]. Notably, EBL + BRZ-treated WT overlapped with EBL alone, indicating that exogenous BR overrides the effects of BRZ [41]. We can nevertheless say that SA has its accumulation inhibited in WT by EBL, but also by BRZ.

In contrast, the *bri1-6* mutant showed no clear response to EBL, confirming the necessity of BRI1 for BR perception. However, BRZ still induced changes in *bri1-6* leaves, demonstrating that BR depletion affects hormone profiles even without canonical BR signaling. This observation aligns with recent suggestions that BRs also function through metabolic or non-transcriptional feedback loops [15], although the mechanisms remain elusive.

The *bri1-6* mutant has been extensively characterized and exhibits classical BR-insensitive phenotypes, including dwarfism, dark green curled leaves, short petioles, and delayed flowering [16,42]. These developmental defects reflect a chronic deficiency in BR perception and are accompanied by alterations in cell expansion, vascular differentiation, and hormone sensitivity. Given these established phenotypes, one might expect that BRZ treatment would induce similar hormonal shifts by reducing BR levels. However, as our data show, the *bri1-6* mutation and BRZ treatments lead to distinct metabolic outcomes, suggesting that long-term developmental remodeling in the mutant and acute BR depletion by BRZ engage partially independent regulatory circuits.

One of the most striking outcomes of our analysis is that the *bri1-6* mutant does not phenocopy BRZ treatment, despite both conditions being associated with reduced BR signaling output. At first glance, one might expect BRZ inhibition of biosynthesis to mimic the effect of a dysfunctional BRI1 receptor, since both ultimately impair BR-dependent transcriptional responses. However, our multivariate analyses, hormone clustering, and direct comparisons reveal divergent and sometimes opposing hormone profiles between the two conditions—particularly in the levels of ABA, JA, and IAA. This discrepancy suggests that BR availability and BR perception act through overlapping but non-identical pathways in shaping hormonal homeostasis. Mechanistically, this uncoupling could reflect several possibilities. First, BRs may have BRI1-independent signaling routes, such as alternate or partially redundant receptor-like kinases (e.g., BRI1-like proteins, BRL1/3), which might remain active in *bri1-6* plants and contribute to residual BR responsiveness [43]. Second, BR levels themselves may serve as metabolic or hormonal cues, influencing the synthesis or catabolism of other phytohormones even in the absence of full BRI1 signaling. For example, BR depletion by BRZ may trigger compensatory changes in JA, SA, and ABA pathways via feedback loops or metabolite sensing mechanisms. In all cases the BR depletion needs to be sensed. Third, *bri1-6* mutants develop under chronic BR signaling deficiency, which could lead to long-term developmental reprogramming and altered hormonal baselines that do not respond to acute treatments in the same way as WT. In contrast, BRZ induces a relatively acute and systemic BR depletion, affecting tissues and pathways differently than the spatially and developmentally constrained receptor mutation.

BRZ is widely used as a pharmacological tool to suppress BR biosynthesis. It is a triazole-type molecule. It binds to DWF4 with a dissociation constant of *Kd* of 1.05 µM [41]. The triazole ring interacts with the heme iron [41]. It possesses high activity with IC50 values of less than 1 μM [21]. It does not directly interfere with GA biosynthesis [44] and has only a minimal inhibitory effect on ABA catabolism [45]. It has been used for the isolation of the *Arabidopsis thaliana* mutants *bzr1-1D* and *bes1-D* [46], thus allowing the identification of transcription factors in the BR signal transduction. Although it appears to be more specific to BR biosynthesis, we cannot exclude the possibility that some of the observed hormone changes—particularly in JA and ABA pathways—may reflect off-target effects. Notably, BRZ still altered hormone profiles in *bri1-6* leaves, suggesting that BR levels alone can trigger regulatory shifts independently of BRI1 perception. However, whether these shifts are strictly due to BR depletion or influenced by broader metabolic interference remains an open question. Future studies using genetic knockouts of BR biosynthesis enzymes (e.g., *dwf4*, *br6ox* mutants) or more specific inhibitors will be essential to confirm the extent to which BRZ effects reflect BR-specific action versus broader metabolic disruption.

The contribution plots highlighted auxin metabolites (e.g., IAA, IAA-Glu) as key drivers of group separation. EBL increased auxin levels in WT, while BRZ reduced them—consistent with prior studies showing synergistic roles of BR and auxin in cell elongation and organ development [47,48]. In *bri1-6* plants, however, IAA was paradoxically elevated. This suggests a compensatory feedback mechanism where BR signaling deficiency leads to upregulation of auxin biosynthesis or reduced conjugation, supporting earlier findings of BR–auxin feedback regulation [49,50].

Cytokinin levels (e.g., iPR, tZR, CZRMP) decreased in response to BRZ, supporting prior findings that BRs promote CK accumulation under optimal conditions [51,52]. Yet, their levels were higher in *bri1-6* plants compared to WT. Glycosylated CK forms like DZR and iP7G showed inverse correlations with active CKs, suggesting possible feedback inhibition or redirection toward storage or transport forms [53]. While BRs have been shown to interact with CKs in regulating meristem activity and leaf growth [54], our results extend this by showing that CK composition is sensitive to both BR levels and perception and may engage in homeostatic compensation via glycosylation pathways [55].

The levels of ABA and its catabolites (PA, DPA) were significantly altered in both WT and *bri1-6* plants during treatments, but the patterns diverged. In WT, BRZ did not significantly affect. Yet, in the *bri1-6* mutant, ABA levels were significantly reduced compared to those in WT. This confirms reports that BRs regulate ABA homeostasis not only through signaling (via BZR1 and ABI5 repression; [31]) but also via metabolic feedback affecting ABA2 and NCED expression [15]. The increase in DPA upon EBL treatment and its decrease with BRZ suggest that BRs promote ABA catabolism, consistent with BR-stimulated DPA production in rice and barley [56,57].

SA levels were lower in *bri1-6* plants, while BzA—its precursor—accumulated, implying that BR signaling may be required for efficient SA biosynthesis from BzA. This supports findings from Choudhary and Senthil-Kumar (2022) [58] showing BR–SA interaction in immune signaling suggests that BRs may fine-tune this interaction by modulating precursor availability.

The levels of JA, JA-Ile, and JA-Me were strongly reduced in *bri1-6* plants compared to WT, confirming prior evidence that BRs are required for jasmonate accumulation under basal conditions [59,60]. BRZ treatment in WT did not affect JA levels, while EBL induced it. In *bri1-6* mutants, BRZ increased JA levels, possibly via BRI1-independent compensation or feedback from altered auxin and CK levels. The observed inverse relationship between JA and its precursor cis-OPDA may indicate an action of BR in the biosynthetic pathway from cis-OPDA to JA. BRs are known to antagonize JA-induced growth inhibition [61], and our findings support a model where BRs buffer JA signaling. This may be part of the general balance of defense versus development.

Across all hormone groups, our data points to a structured co-regulation system where BRs act not only as growth promoters but as central modulators of hormonal homeostasis. The co-regulation patterns in the correlation matrix (Figure 2B) are closely mirrored by the hierarchical clustering of hormone abundances (Figure 2A). Stress-associated hormones (SA, JA, JA-Ile, JA-Me, and ABA), together with iP7G and DZR, form a stable Group 1 cluster in both panels. Auxin-related metabolites (IAA, IAA-Asp, IAM, IAA-GE, IAA-Glu, PAA, and PAAM) constitute a coherent Group 3 in both analyses. Importantly, cis-OPDA and BzA co-cluster within Group 2 (with PA, DPA, and cytokinin derivatives) in both Figure 2A and Figure 2B, underscoring the robustness of this ABA/CK-linked module. Thus, despite the different underlying metrics (abundance vs. correlation), the three hormone modules are conserved across both representations, reinforcing the biological validity of our network partitioning. We can nevertheless point at the specificity of SA. It belongs to Group 1; this is correlated to ABA and JA. It is yet clearly negatively correlated with Group 3 hormones, such as IAA and its conjugates, contrary to the other Group 1 hormones.

The three main hormone clusters identified—stress (Group 1), growth/differentiation (Group 2), and auxin-driven (Group 3)—interact dynamically. Negative correlations between stress and growth hormones support the long-standing model of growth–defense trade-offs [62]. It is important to note that these clusters reflect co-variation in hormone accumulation, not necessarily convergence in regulatory output. On the contrary, hormones that cluster together may trigger divergent or even antagonistic responses, depending on context. For instance, SA and JA consistently co-cluster in both our dataset and previous studies (e.g., [63]), yet they often act antagonistically in plant defense, mediating responses to biotrophic versus necrotrophic pathogens. Such coordinated accumulation coupled with divergent signaling output likely represents a fine-tuning mechanism that allows plants to balance overlapping developmental or stress cues.

## 4. Materials and Methods

### 4.1. Plant Materials and Growth Conditions

*Arabidopsis thaliana* seeds of the WT (ecotype Col-1) and *bri1-6* mutant were obtained from the NASC European Arabidopsis Stock Centre. Seeds were surface sterilized by soaking in a solution of 5% sodium hypochlorite (NaOCl), 10% ethanol (EtOH), and 0.025% Tween 20 for 15 min, followed by rinsing five times with sterile water, as described by [64]. Seeds were then germinated and grown on solid sterile ½ strength MS medium supplemented with EBL and/or BRZ at the indicated final concentrations. Plants were grown at a constant temperature of 22 °C in an artificial climate chamber (14 h light: 10 h dark cycle) under white light provided by continuous wide-spectrum LEDs (Philips, Amsterdam, The Netherlands) at an intensity of 150 μmol m^−2^ s ^−1^. For phytohormone analysis, plants were grown for 21 days, and 100 mg leaf samples were harvested.

### 4.2. Preparation of Chemical Stocks

24-EBL was dissolved in EtOH to prepare stock solutions of 10 M or 100 µM. BRZ was dissolved in EtOH to obtain a 10 mM stock solution. These stock solutions were added to sterile ^1^/_2_ MS medium to achieve final concentrations of 10 nM for EBL and 1 µM for BRZ. The final EtOH concentration in the growth medium was the same across all variants and did not exceed 0.01%.

### 4.3. Hormone Extraction and Quantification

Frozen samples (100 mg FW) were homogenized using liquid nitrogen in a mortar and pestle. Phytohormones were extracted using a cold (−20 °C) methanol/water/formic acid mixture (15/4/1, *v*/*v*), as described by [65]. Isotope-labeled standards (10 pmol/sample) were added to the samples: ^13^C_6_-IAA, ^2^H_4_-OxIAA, ^2^H_4_-OxIAA-GE (Cambridge Isotope Laboratories, Tewksbury, MA, USA); ^2^H_4_-SA, ^2^H_2_-GA_19_ (Sigma-Aldrich, Burlington, MA, USA); ^2^H_3_-PA, ^2^H_3_-DPA (NRC-PBI); ^2^H_6_-ABA, ^2^H_5_-JA, ^2^H_5_-tZ, ^2^H_5_-tZR, ^2^H_5_-tZRMP, ^2^H_5_-tZ7G, ^2^H_5_-tZ9G, ^2^H_5_-tZOG, ^2^H_5_-tZROG, ^15^N_4_-cZ, ^2^H_3_-DZ, ^2^H_3_-DZR, ^2^H_3_-DZ9G, ^2^H_3_-DZRMP, ^2^H_7_-DZOG, ^2^H_6_-iP, ^2^H_6_-iPR, ^2^H_6_-iP7G, ^2^H_6_-iP9G, ^2^H_6_-iPRMP (Olchemim, Olomouc, Czech Republic). The extracts were passed through reversed-phase cation-exchange solid-phase extraction (SPE) columns (Oasis-MCX, Waters, Milford, MA, USA) in mixed-phase cation-exchange mode. The hormone fraction, containing ABA, IAA, SA, and JA, was eluted with methanol. Hormone metabolites were analyzed by HPLC (Ultimate 3000, Dionex, Sunnyvale, CA, USA) coupled with a hybrid triple quadrupole/linear ion trap mass spectrometer (3200 Q TRAP, Applied Biosystems, Foster City, CA, USA). Hormone quantification was performed using the isotope dilution method with multilevel calibration curves (R^2^ > 0.99). Data processing was carried out with Analyst 1.5 software (Applied Biosystems).

### 4.4. Statistical Analysis

#### 4.4.1. Analysis of Variance (ANOVA) and Post Hoc Tests

For each metabolite, a two-way ANOVA was performed to assess the effects of genotype, treatment, and their interaction. When ANOVA indicated significant effects (*p* < 0.05), pairwise comparisons were conducted using Fisher’s Least Significant Difference (LSD) post hoc test. Statistical grouping was visualized using boxplots, where groups sharing the same letter are not significantly different (α = 0.05). All statistical analyses were performed in Python 3.11.x using the scipy.stats and statsmodels libraries.

#### 4.4.2. Boxplots

Boxplots were generated to visualize the distribution of metabolite levels across treatments and genotypes. Each boxplot represents the median, interquartile range, and potential outliers for four biological replicates per condition. Statistical groupings (letters) were assigned based on pairwise *t*-tests (Fisher’s LSD) and annotated above each boxplot.

#### 4.4.3. Principal Component Analysis (PCA)

PCA was used to explore the overall variability in metabolite profiles. Data were centered and scaled (unit variance) before PCA. The number of principal components (PCs) was determined based on the explained variance ratio. PCA biplots were generated to visualize the separation of samples by genotype and treatment, with metabolites contributing to the PCs represented as vectors. PCA was performed using the Scikit-learn library in Python.

#### 4.4.4. Heatmap

A heatmap was constructed to visualize the correlation matrix of metabolite levels across all samples. Pearson correlation coefficients were calculated, and hierarchical clustering was applied to group metabolites with similar profiles. The heatmap was generated using the Seaborn library, with a color gradient representing correlation strength.

#### 4.4.5. Software and Code Availability

All analyses were performed using custom Python scripts, which are available upon request. The following key libraries were used: pandas for data manipulation, scipy.stats and statsmodels for statistical tests, Scikit-learn for PCA, and Seaborn 0.13.2 and matplotlib for visualization.

## 5. Conclusions

This study demonstrates that BR biosynthesis and BR perception exert differential and sometimes opposing effects on the hormone landscape in *Arabidopsis thaliana*. While BRI1-mediated signaling is crucial for many aspects of hormonal regulation, our data show that BR availability also independently influences hormone profiles, even in the absence of functional BR perception. The *bri1-6* mutant does not phenocopy BRZ treatment, indicating that BRs function not only through canonical receptor pathways but may also modulate plant physiology through broader metabolic and feedback mechanisms. These findings challenge the assumption that BRZ treatment is equivalent to BR-insensitive mutants and highlight the importance of considering both signaling and hormone levels in plant stress and growth regulation. By dissecting BR-dependent and BR-independent layers of hormone control, our study provides a more nuanced understanding of how BRs shape the dynamic balance between growth and defense, with implications for crop improvement and stress resilience strategies.

## Data Availability

Data is available on request from the corresponding author.

## Supplementary Materials

The following supporting information can be downloaded at: https://www.mdpi.com/article/10.3390/ijms26199644/s1.

## Abbreviations

ABA	Abscisic Acid
ABA-GE	Abscisic Acid Glucose Ester
BAK1	Brassinosteroid-Insensitive 1-Associated Receptor Kinase 1
BES1	Brassinosteroid-Insensitive 1-EMS-Suppressor 1
BzA	Benzoic Acid
BR	Brassinosteroids
BRZ	Brassinazole (Brassinosteroid Biosynthesis Inhibitor)
BZR1	Brassinosteroid-Resistant 1
BRI1	Brassinosteroid Insensitive 1 Receptor Kinase
BR6ox	Brassinosteroid-6-Oxidase
DPA	Dihydrophaseic Acid
DZR	Dihydrozeatin Riboside
EBL	24-Epibrassinolide
JA	Jasmonic Acid
JA-Ile	Jasmonic Acid-Isoleucine
JA-Me	Jasmonic Acid Methyl Ester
IAA	Indole-3-Acetic Acid
IAA-Asp	Indole-3-Acetic Acid Aspartate
IAA-GE	Indole-3-Acetic Acid Glucose Ester
IAA-Glu	Indole-3-Acetic Acid Glutamine
IAM	Indole-3-Acetamide
iP	Isopentenyl Adenine
iP7G –	Isopentenyl Adenine-7-Glucoside
iP9G	Isopentenyl Adenine-9-Glucoside
iPR	Isopentenyl Adenine Riboside
MeS-ZR	2-Methylthio Zeatin Ribosides of Trans-Zeatin
PCA	Principal Component Analysis
PA	Phaseic Acid
PAA	Phenylacetic Acid
PAA-M	Phenylacetamide
SA	Salicylic Acid
tZR	Trans-Zeatin Riboside
tZ	Trans-Zeatin
tZRMP	Trans-Zeatin Riboside Monophosphate
ZR	Zeatin Riboside
