# Supplementary material for: Brassinosteroid Synthesis and Perception Differently Regulate Phytohormone Networks in Arabidopsis thaliana
Source: Int J Mol Sci. 2025 Oct 2;26(19):9644. doi: 10.3390/ijms26199644 (PMC12524406; doi:10.3390/ijms26199644)
Supplement: Supplementary file 1 [file ijms-26-09644-s001.zip › ijms-3831011-supplementary.pdf]

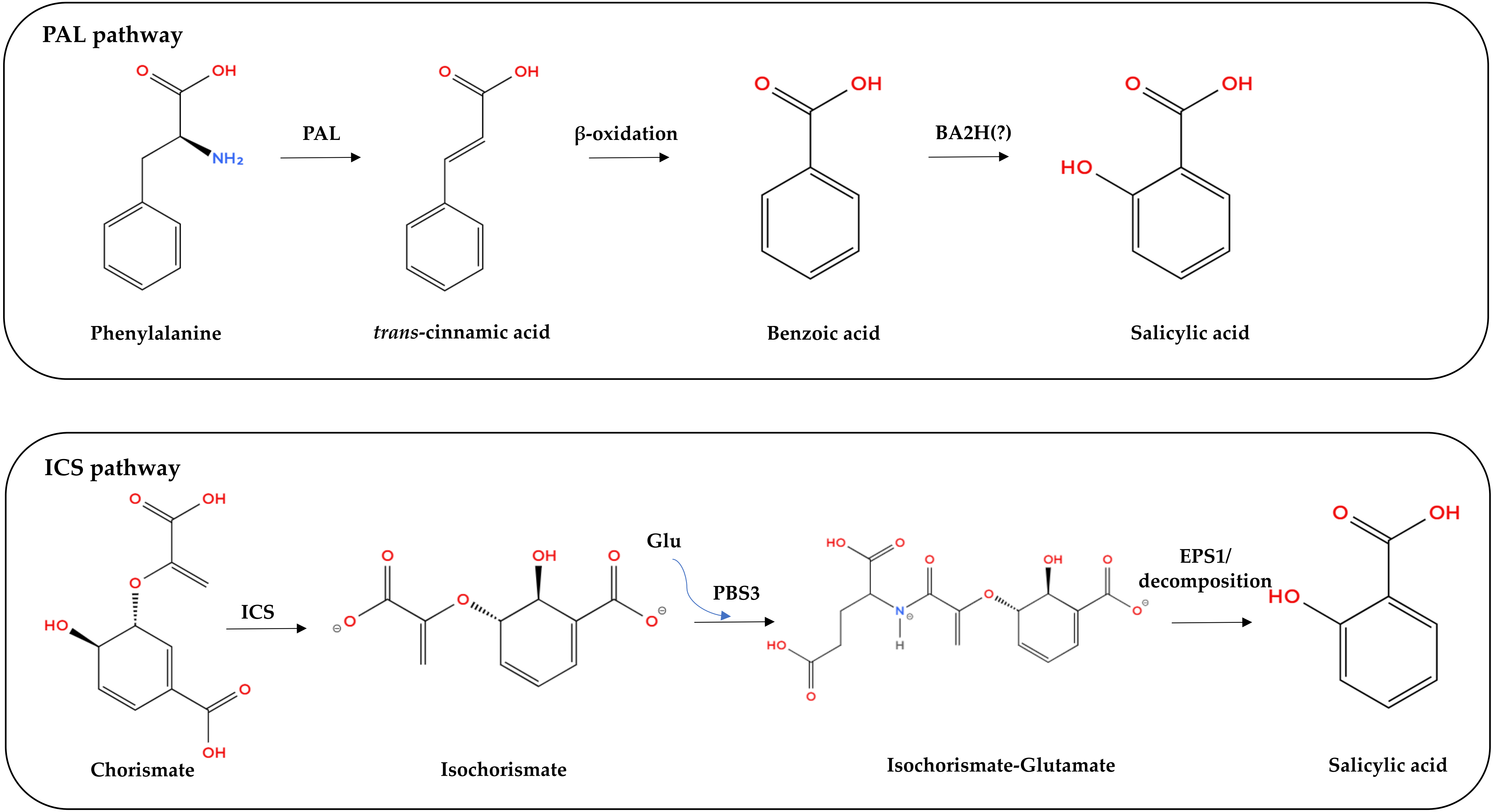
PAL pathway
PAL
β-oxidation
BA2H(?)
Phenylalanine
trans-cinnamic acid
Benzoic acid
Salicylic acid
ICS pathway
ICS
Glu
PBS3
EPS1/
decomposition
Chorismate
Isochorismate
Isochorismate-Glutamate
Salicylic acid

PAL pathway. SA biosynthesis through the phenylalanine ammonia-lyase (PAL) pathway. Phenylalanine is deaminated by PAL to yield *trans*-cinnamic acid, which is subsequently converted into benzoic acid (BA) through β-oxidation. Benzoic acid is then assumed to be hydroxylated by putative benzoic acid 2-hydroxylase (BA2H) to produce SA.

ICS pathway. In *Arabidopsis thaliana* and other crucifers, SA is predominantly synthesised via the ICS pathway, where ICS converts chorismate into isochorismate, followed by a two-step transformation into SA mediated by PBS3 and EPS1 (or spontaneously decomposes).

Huang J, Van Der Hoorn RAL. The ancestral salicylic acid biosynthesis pathway in plants. Trends Plant Sci. 2025 Aug;S1360138525002304.

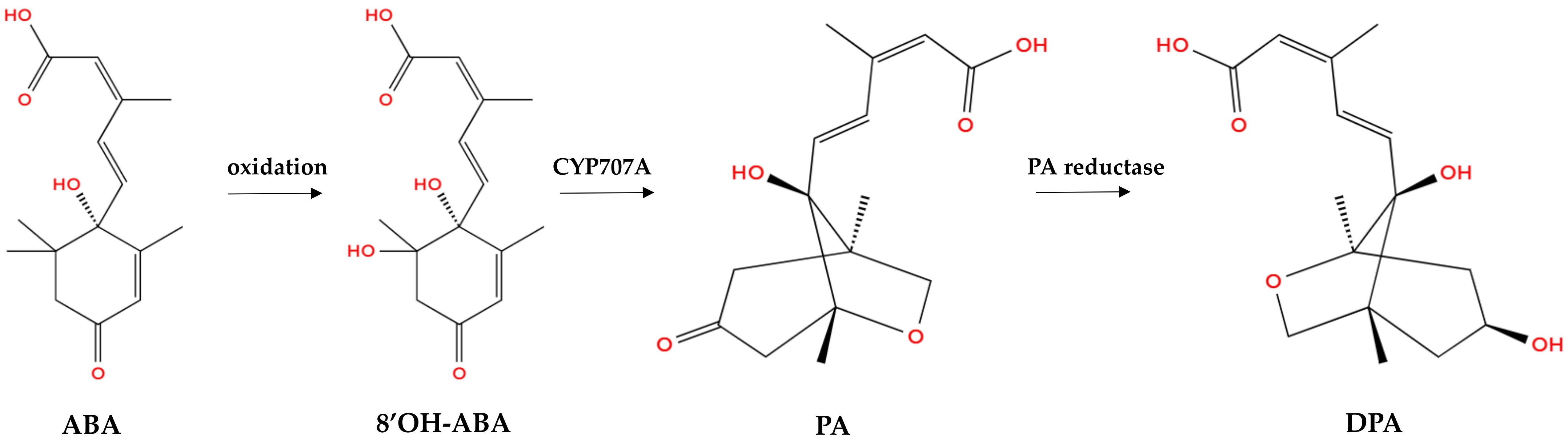

Catabolic pathway for ABA.
ABA, abscisic acid; 8′OH-ABA, 8′-hydroxyABA; PA, phaseic acid; DPA, dihydrophaseic acid

Priest DM, Ambrose SJ, Vaistij FE, Elias L, Higgins GS, Ross ARS, et al. Use of the glucosyltransferase UGT71B6 to disturb abscisic acid homeostasis in *Arabidopsis thaliana*. Plant J. 2006 May;46(3):492–502.

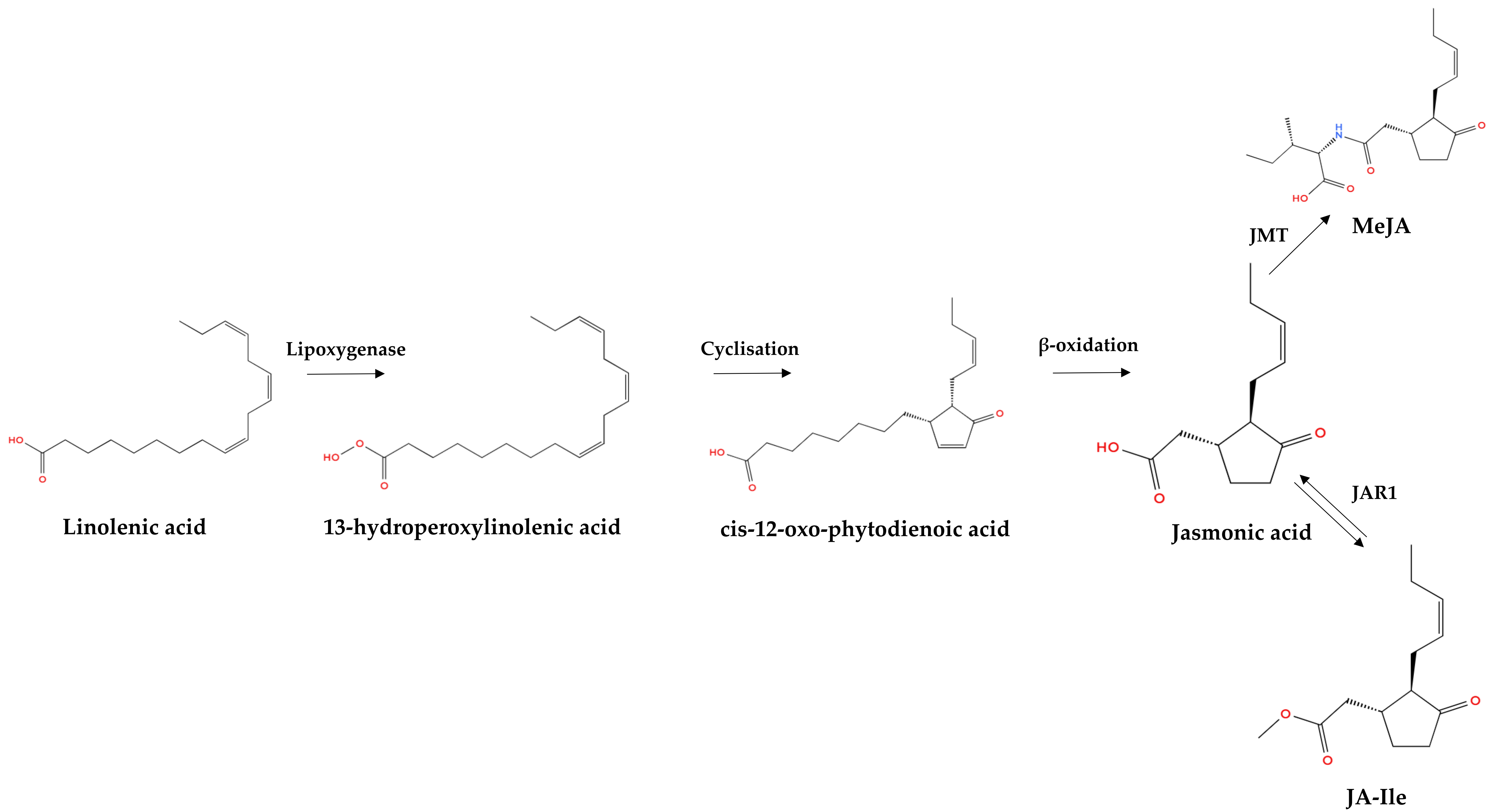
Lipoxygenase
Cyclisation
β-oxidation
JMT
JAR1
HO
O
H
N
Linolenic acid
13-hydroperoxylinolenic acid
cis-12-oxo-phytodienoic acid
Jasmonic acid
MeJA
JA-Ile

Schematic diagram of jasmonic acid biosynthesis. In the chloroplast, JA biosynthesis begins with the chloroplast membrane release of linolenic acid, which is finally converted to 12-oxo-PDA. Upon transport of 12-oxo-PDA into the peroxisome, a series of enzymes work to convert it to JA, which is then exported to the cytoplasm. JA may be metabolized into different compounds depending on the chemical modification of the carboxylic acid group, the pentenyl side chain, or the pentanone ring.

JMT, jasmonic acid carboxyl methyltransferase; MeJA, methyl jasmonate; JAR1, jasmonate amino acid synthetase 1; JA-Ile, jasmonyl isoleucine.

Ali MdS, Baek KH. Jasmonic Acid Signaling Pathway in Response to Abiotic Stresses in Plants. Int J Mol Sci. 2020 Jan 17;21(2):621.

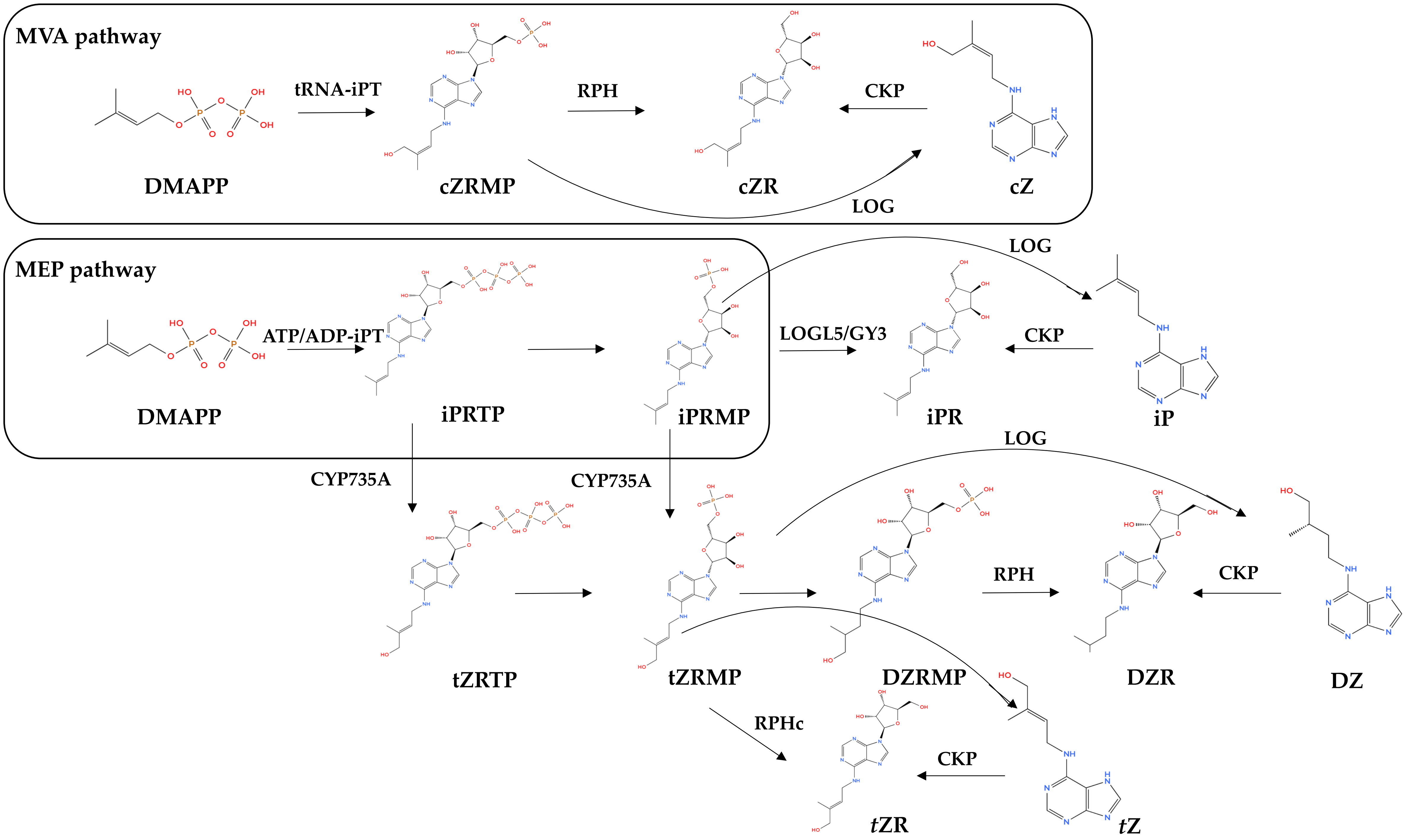

MVA pathway
DMAPP
tRNA-iPT
cZRMP
RPH
cZR
CKP
cZ
LOG
MEP pathway
DMAPP
ATP/ADP-iPT
iPRTP
iPRMP
LOG
LOGL5/GY3
iPR
CKP
iP
LOG
CYP735A
CYP735A
tZRTP
tZRMP
DZRMP
RPH
DZR
CKP
DZ
RPHc
tZR
CKP
tZ

Biosynthesis of iP- and cZ-type cytokinin nucleotides is derived from the methylerythritol phosphate (MEP) and mevalonate (MVA) pathways in the plastids and cytoplasm, respectively. iP-type cytokinin nucleotides are synthesized in the plastids and are transported first to the endoplasmic reticulum (ER) and finally to the cytoplasm, where various cytokinin derivatives are produced by LOG enzymes. Cytokinin/purine riboside nucleosidase 1 (CPN1) is localized in the cell walls, where it catalyzes deribosylation of the cytokinin ribosides cZR, cZROG, and tZR, producing the cytokinin free bases cZ, cZOG, and tZ in the apoplast.

IPT, adenylate isopentenyl transferase; CYP735A, cytochrome P450 monooxygenase, family 735, subfamily A; CKP, cytokinin riboside phosphorylase; LOG, cytokinin phosphoribohydrolase LONELY GUY; LOGL5/GY3, cytokinin phosphoribohydrolase LONELY GUYLIKE 5/quantitative trait loci GY3; RPH, 50-ribonucleotide phosphohydrolase.

Zhao J, Wang J, Liu J, Zhang P, Kudoyarova G, Liu CJ, et al. Spatially distributed cytokinins: Metabolism, signaling, and transport. Plant Commun. 2024 July;5(7):100936.

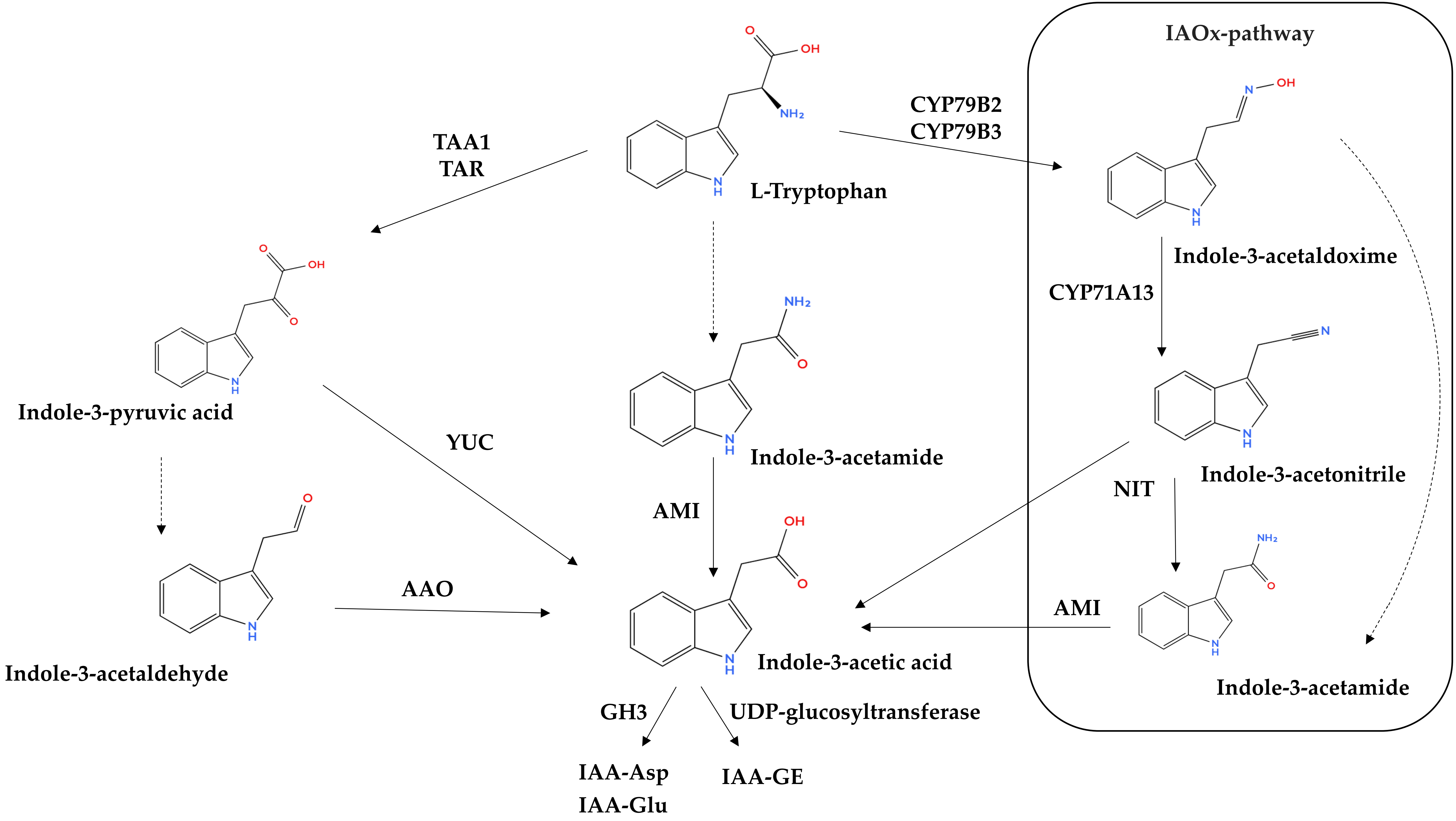

IAOx-pathway
L-Tryptophan
TAA1
TAR
CYP79B2
CYP79B3
Indole-3-acetaldoxime
CYP71A13
Indole-3-acetonitrile
NIT
Indole-3-acetamide
AMI
Indole-3-pyruvic acid
YUC
Indole-3-acetamide
AMI
Indole-3-acetaldehyde
AAO
Indole-3-acetic acid
GH3
UDP-glucosyltransferase
IAA-Asp
IAA-Glu
IAA-GE

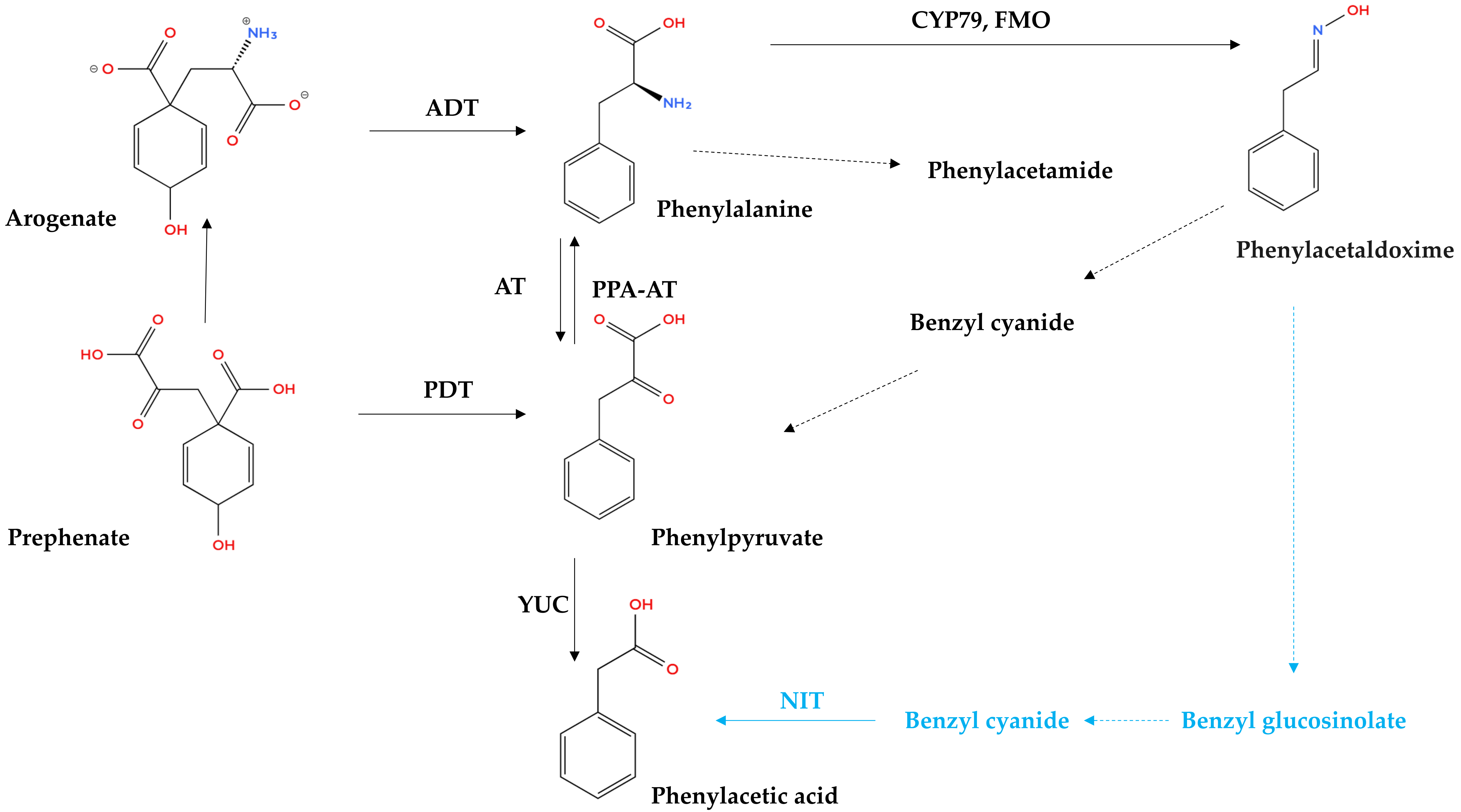

Arogenate
ADT
Phenylalanine
CYP79, FMO
Phenylacetaldoxime
Phenylacetamide
AT
PPA-AT
Benzyl cyanide
Prephenate
PDT
Phenylpyruvate
YUC
Phenylacetic acid
NIT
Benzyl cyanide
Benzyl glucosinolate

1. Proposed pathways of L-tryptophan-dependent IAA biosynthesis in plants. The IAOx-pathway that is seemingly restricted to indole glucosinolate-producing plant species is given in the box. Dashed lines indicate assumed reaction steps for which the corresponding enzymes have yet to be identified.

2. Schematic diagrams of PAA biosynthesis. Blue arrows and metabolites represent pathways and metabolites present only in Brassicales species.
IAA, indole-3-acetic acid, AT, amino transferase; CYP79, cytochrome P450 mono-oxygenase of the 79 family; FMO, flavin-containing mono-oxygenase; GH3, Gretchen Hagen 3 auxin-amido synthetase; IAMT, IAA carboxymethyltransferase; NIT, nitrilase; PAT, prephenate aminotransferase; PDT, prephenate dehydratase; PPA-AT, phenylpyruvate aminotransferase; TAA, tryptophan aminotransferase of Arabidopsis; TS, tryptophan synthase; UGT, UDP-glucuronosyltransferase; YUC, YUCCA family of flavin-containing mono-oxygenase; VAS; methionine aminotransferase.

Sánchez-Parra B, Frerigmann H, Alonso MM, Loba V, Jost R, Hentrich M, et al. Characterization of Four Bifunctional Plant IAM/PAM-Amidohydrolases Capable of Contributing to Auxin Biosynthesis. Plants. 2014 Aug 7;3(3):324–47.

Perez VC, Zhao H, Lin M, Kim J. Occurrence, Function, and Biosynthesis of the Natural Auxin Phenylacetic Acid (PAA) in Plants. Plants. 2023 Jan 6;12(2):266.
